# Deformation and Failure of MXene Nanosheets

**DOI:** 10.3390/ma13051253

**Published:** 2020-03-10

**Authors:** Daiva Zeleniakiene, Gediminas Monastyreckis, Andrey Aniskevich, Paulius Griskevicius

**Affiliations:** 1 Department of Mechanical Engineering, Kaunas University of Technology, 51424 Kaunas, Lithuania; gediminas.monastyreckis@ktu.edu (G.M.); paulius.griskevicius@ktu.lt (P.G.); 2 Institute for Mechanics of Materials, University of Latvia, LV-1004 Riga, Latvia; andrey.aniskevich@pmi.lv

**Keywords:** MXene, mechanical behavior, finite element modeling

## Abstract

This work is aimed at the development of finite element models and prediction of the mechanical behavior of MXene nanosheets. Using LS-Dyna Explicit software, a finite element model was designed to simulate the nanoindentation process of a two-dimensional MXene Ti_3_C_2_T_z_ monolayer flake and to validate the material model. For the evaluation of the adhesive strength of the free-standing Ti_3_C_2_T_z_-based film, the model comprised single-layered MXene nanosheets with a specific number of individual flakes, and the reverse engineering method with a curve fitting approach was used. The interlaminar shear strength, in-plane stiffness, and shear energy release rate of MXene film were predicted using this approach. The results of the sensitivity analysis showed that interlaminar shear strength and in-plane stiffness have the largest influence on the mechanical behavior of MXene film under tension, while the shear energy release rate mainly affects the interlaminar damage properties of nanosheets.

## 1. Introduction

A new class of two-dimensional (2D) nanomaterials, MXenes, was discovered in the last decade [1]. MXenes are transition metal carbides or nitrides produced by the etching of the A element from the MAX phases. Typically, nanomaterials can be divided into two groups: hydrophilic but not conductive, such as transition metal oxides or clays; and conductive but not hydrophilic, such as graphene. However, some MXenes (Ti_2_CT_z_, Ti_3_C_2_T_z_) have the unique characteristics of both groups. Due to the combination of the electrical conductivity of transition metal carbides and the hydrophilicity of hydroxyl or oxygen-terminated surfaces, these MXenes behave as “conductive clays” [2].

MXenes have been widely investigated during the past few years. The main interest has been directed at the electric properties, their applications for sensors, energy storage (batteries, supercapacitors, hydrogen evolution reaction catalysts), harvesting, electromagnetic shielding, tribology, etc. [3,4,5,6]. Pioneer studies of the mechanical properties of MXenes showed promising results [1,2,7]. Elastic properties were obtained experimentally by nanoindentation with the tip of an atomic force microscope (AFM) and the elastic modulus of the most investigated MXene material, Ti_3_C_2_T_z_, was obtained at 0.33 ± 0.03 TPa [8]. According to classical molecular dynamics simulation [7], which does not take into account various material defects, the modulus was higher and equal to 0.502 TPa.

MXenes exhibit a high bending stiffness [9]. The critical deformations are much higher than the graphene ones, and this is an important feature of flexible electronics [4,10,11]. MXenes have good interactions with polymeric matrices for polymer composite applications [4,12,13]. For these reasons, MXene could be a good candidate to provide electrical conductivity for fiber-reinforced plastic composites without losing desirable mechanical properties and imparting additional self-sensing functions. While some of the mechanical properties of MXene have been determined theoretically or experimentally, the mechanical behavior of MXene, and particularly its failure mechanisms, has not been studied well, and there is a huge lack of data that are needed for the development of polymer composites filled with MXene 2D nanosheets.

The aim of this study was to investigate the micromechanical behavior of MXene nanosheets by developing finite element (FE) computational models. The objectives were to (1) develop an FE model composed of single-layered MXenes nanosheets with a specific number of individual flakes for explicit analysis; and (2) identify the material parameters by FE simulation of interface shearing. The novelty of this study is that it shows first insights into the deformation and failure mechanisms of this new nanomaterial, as well as providing a basis for the future design of polymer composites reinforced with MXene nanosheets, and the development of MXene–polymer coatings with high-density MXene–MXene interactions.

## 2. Modeling Methods

### 2.1. FE Model of Nanoindentation

**Figure 1:**
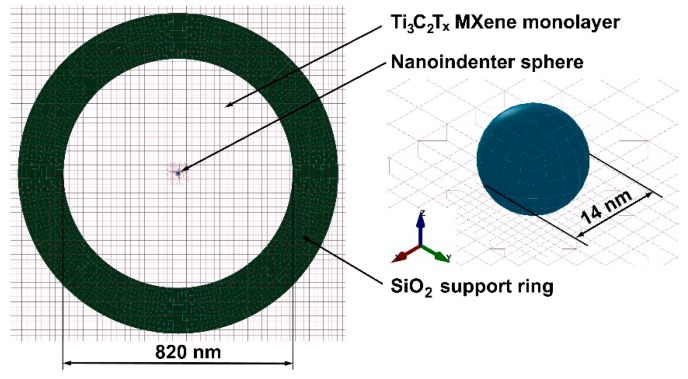
Finite element (FE) model for the nanoindentation process analysis.

The main purpose of this presimulation was to build an explicit FE model of a Ti_3_C_2_T_z_ MXene monolayer flake for analysis of the nanoindentation process in LS-Dyna software and, using the force vs. deflection curve, validate the FE model and material characteristics. The FE model (Figure 1) was developed according to the experimental data presented by Lipatov et al. [8], where it was considered that the MXene flake has isotropic properties, and therefore, the membrane can be parametrized using Young’s modulus, *E* and Poisson’s ratio, *v*.

The thickness of the Ti_3_C_2_T*_z_* monolayer flake is an important parameter as it influences the results of the nanoindentation experiments. Using AFM for the determination of the thickness of monolayers of 2D materials has some limitations. The Ti_3_C_2_T*_z_* MXene flake thicknesses obtained by AFM can differ significantly [8,14], and this directly affects the determined value of Young’s modulus. MXene flakes with a thickness of 0.98 nm were modeled [1,2,11]. The mechanical properties of MXenes and the SiO_2_ support ring used in the model are based on the analysis of the literature data. The indenter was a diamond crystal with a modulus of 1 TPa. The mechanical properties of the materials used in the nanoindentation simulation are presented in Table 1.

The 2D Ti_3_C_2_T_z_ MXene flake was modeled with shell elements using a linear elastic material model. The size of the shell elements was 10 nm, while the center of the monolayer (contact zone with nanoindenter) was decreased to as little as 1.25 nm. The nanoindenter was defined as an elastic solid sphere (diameter 14 nm). The bottom of the MXene flakes was supported on the ring surface of SiO_2_, which was fully fixed. Two pinball-type contacts (AUTOMATIC_NODES_TO_SURFACE) were used: surface of SiO_2_—bottom flake, and surface of the nanoindenter—top flake. Two simulations were performed with initial pretension and without. As it was an explicit analysis, the initial pretension initiated the oscillation of the flake; therefore, the *CONTROL_DYNAMIC_RELAXATION must be activated.

The indentation was described by the dependence of the displacement on time. By linearly increasing the displacement, which gives a constant velocity, a small impact phenomenon was obtained in the model, and this initiated the vibrations of the MXene monolayer flake. Finally, a smooth increase in the displacement was chosen based on the assumption that the average speed of the nanoindenter is equal to ~1 m/s, and the speed increases linearly from 0 up to the 2v_avg. The loading curve displacement vs. time was obtained by the function u(t)=∫v(t)dt.

A methodology for deriving the material parameters from experimental results, known as parameter identification, was applied here using the optimization procedure. The same nanoindentation FE model without initial pretension has been chosen to evaluate the sensitivity of the material and the geometric parameters to the mechanical behavior of the sample. The Young’s modulus and flake thickness were chosen as variables. The Young’s modulus varied between 200 and 400 GPa and the thickness from 0.4 to 1.5 nm. The mean square error was used as a curve fitting metric. The experimental force, *F* vs. deflection δ curve can be described by the following equation [8]:

(1) $$F=\sigma_{0}^{2D}\pi \delta +E^{2D}\frac{q^{3}\delta^{3}}{r^{2}},$$

where $\sigma_{0}^{2D}=\frac{\sigma }{h}$ represents the prestress in the membrane, $E^{2D}=\frac{E}{h}$ is the 2D elastic modulus (thickness h=0.98 nm), and *r* is the radius of the well [8]. The dimensionless constant, *q*, is related to *ν* as $q=1/\left(1.049-0.15\nu -0.16\nu^{2}\right)=0.9933.$ The first term in Equation (1) corresponds to the prestretched membrane regime and is valid for small loads. The second term for the nonlinear membrane behavior is characterized by a cubic *F*~δ^3^ relationship with a coefficient of *E^2D^*, which dominates at large loads. For comparison, only the second part of Equation (1) was used; therefore, the prestretching was not taken into account during the simulation.

### 2.2. FE Model of Pure MXene Film

The stability of the stacked two-dimensional transition metal carbides and their interlayered friction in different configurations are comparatively studied by means of density functional theory. In recent years, the adhesive interactions of monolayers and few-layer 2D materials have been intensively investigated. At equilibrium, a larger interlayer distance corresponds to a smaller binding energy, suggesting an easier sliding between the layers [17]. Nanoindentation has been widely used to characterize the adhesion of thin films [18,19,20]. One of the important questions is to understand how the properties—in particular, adhesive strength—change when transitioning from bulk to 2D forms of the material. However, to our knowledge, no theoretical or experimental studies of the adhesive properties of MXenes have been reported to date. The results of direct AFM measurements of adhesion of two MXenes, Ti_3_C_2_T*_z_* and Ti_2_CT*_z_*, with a SiO_2_-coated Si spherical tip is one of the recent studies of adhesion properties [17].

**Figure 2:**
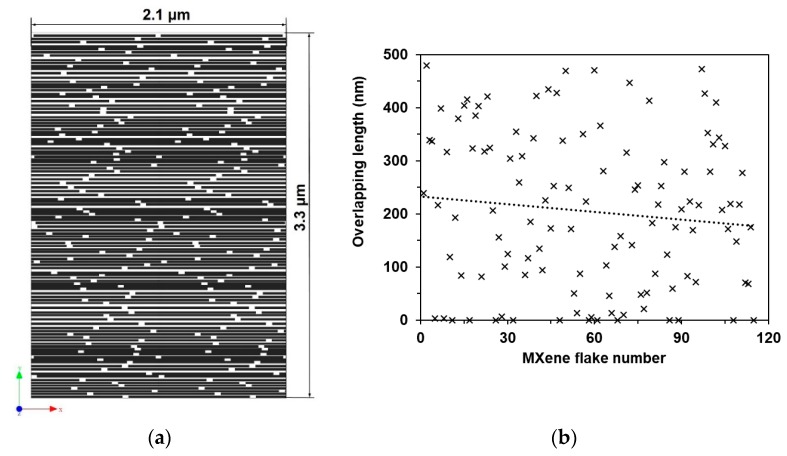
Estimation of nanosheets overlapping: (**a**) segment of the Digimat model of free-standing Ti_3_C_2_T_z_ film, (**b**) obtained overlapping length distribution by simulation of randomly placed nanosheets.

One of the ways to analyze adhesive strength is to apply the reverse engineering method. Assuming that adhesion energy between surfaces of free-standing MXene nanosheets exists [21], then the strength of the interlayer surface has to depend on the overlapping area. For the study of adhesive interactions, the experimental tensile test data of the assembled free-standing Ti_3_C_2_T_z_-based films [4] were used. It was assumed that the single-layered nanosheet has a square form of 1 μm length [22,23,24,25], consisting of 18 Ti_3_C_2_T*_z_* individual flakes and an average thickness of 20 nm [25]. In total, 164 layers of single-layered nanosheets per film of 3.3 μm thickness were used in experimental testing [4]. As the overlapping of nanosheets has an essential influence on the strength of the interlayer surface, a 2D analysis of randomly placed rectangle nanosheets was performed using materials modeling software, Digimat. For the overlapping analysis, MXene nanosheets with dimensions of 1000 × 20 nm were chosen. The overall thickness of MXene film formed from these nanosheets was 3.3 μm, as it was in testing [4] (Figure 2a). The results show that the average overlapping of nanosheets is 20% (Figure 2b). This overlap value was used to create the FE model.

**Figure 3:**
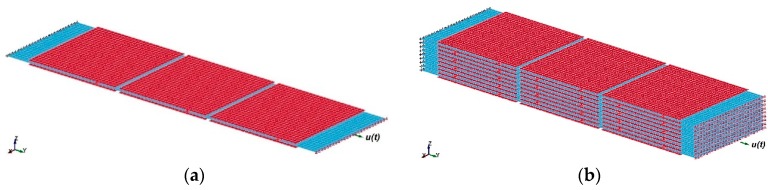
FE model for the simulation of MXene nanosheet interface shear strength with 20% overlapping length: (**a**) 3-nanosheets-thick model, (**b**) 19-nanosheets-thick model. Red and blue colors are used for contrast to better show the single-layered nanosheets.

For FE simulation of the interface shear strength of MXene nanosheets (1000 nm × 1000 nm × 20 nm), three- (Figure 3a) and nineteen- (Figure 3b) layer models were developed with an overlapping length of 200 nm (20%).

**Figure 4:**
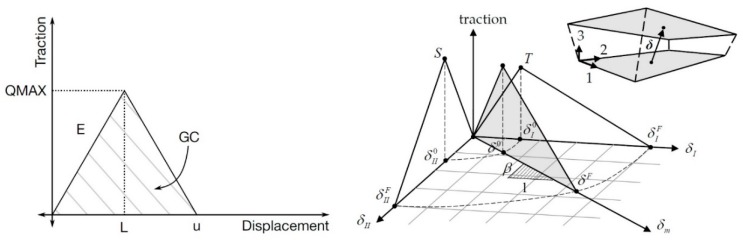
Bilinear traction–separation and the mixed-mode traction–separation law [26].

The FE model was designed taking into account the experimental setup data presented in the study [4]. The single-layered Ti_3_C_2_T_z_ MXene nanosheet was modeled with shell elements. The Young’s modulus of the MXene nanosheet was set at 333 GPa; Poisson’s ratio—0.227; the size of shell elements—5 nm. The nodes on the left-side edges had a fixed 4 degrees-of-freedom, allowing free contraction in the *x*-direction and rotation about the *z*-axis. For the right-side nodes, the displacement vs. time *u*(*t*) was applied with a speed of 1 mm/ms. The interface between the single-layered nanosheets was modeled using tiebreak contact, *CONTACT_AUTOMATIC_ONE_WAY_SURFACE_TO_SURFACE_TIEBREAK. The discrete crack model with power-law damage, which works with offset shell elements (option = 11), shown in Figure 4, was chosen. The parameters needed to describe tiebreak contact are presented in Table 2.

The internal force acting per nanosheet was calculated from the experimentally determined [4] tensile strength value as follows,

(2) $$F_{1L}=\sigma_{u}hw,$$

where $\sigma_{u}$—experimentally determined [4] ultimate stress; h—nanosheet thickness; w—nanosheet width. $F_{1L}=\sigma_{\max}hw=22\left(\mathrm{Mpa}\right)\times 20\left(\mathrm{nm}\right)\times 1\left(\mathrm{nm}\right)=440.0\;\mathrm{nN}$.

The strength of the assembled free-standing Ti_3_C_2_T_z_-based film depends on the nanosheet interface shear strength,

(3) $$\tau_{u}=\frac{F_{1L}}{OVL\times L\times w}=\frac{\sigma_{u}h}{OVL\times L}$$

where OVL —overlapping coefficient; L—nanosheet length; $\tau_{u}=\frac{22\;(\mathrm{MPa})\times 20\;(\mathrm{nm})}{0.2\times 1000\;(\mathrm{nm})}=2.2\;\mathrm{MPa}$.

The minimum interlayer strength value achieved by an average overlapping length of 20% satisfies the experimental results [4]. For an FE model of three layers of MXene nanosheets $L_{\mathrm{ovl}}=2200\;\mathrm{nm}$, the maximum resultant load acting on MXenes nanosheet cross-sections is the following:

(4) $$F_{\max}=\tau_{u}\left(\mathrm{Mpa}\right)\times n_{l}\times OVL\times L\times w=2.2\times 2\times 0.2\times 1000\left(\mathrm{nm}\right)\times 1000\left(\mathrm{nm}\right)=880\;\mathrm{nN}$$

where$\;n_{l}$—the number of interfaces between three nanosheets.

This value was used as the criteria to validate the FE model for interface shear strength simulation. The graphical optimization tool LS-OPT was used for the identification of material constants. The material parameters were obtained using the curve fitting approach with the parameterized simulation of the physical tests and calibration to the test results. The objective was to minimize the mean squared error between the test results [4] and the FE simulation results.

**Figure 5:**
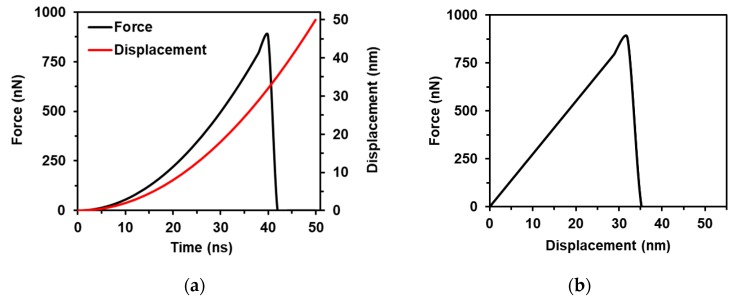
Dependencies for FE modeling: (**a**) displacement vs. time curve used for loading (red) and force vs. time for the curve fitting procedure (black); (**b**) force vs. displacement curve recalculated from the experiments [4].

The loading was described by a curve of linearly increased displacement vs. time. As was mentioned before, this gives a constant velocity, which initiates vibration of the structure and makes its behavior uncertain. Therefore, in the second step, a smooth increase of displacement was chosen for loading, from the assumption that an average speed of tensile loading is equal to ~1 m/s, and speed is increasing linearly from 0 up to the $2v_{avg}$. The loading curve, displacement versus time, was obtained by integrating the linearly increasing velocity function: u(t)=∫v(t)dt (Figure 5a). The response F(t) was used for the curve fitting procedure and, for FE model validation, it was transformed into the F(u) curve (Figure 5b). Using Equation (2), the experimental tensile curve [4] was recalculated into a force versus displacement curve and used for model validation.

## 3. Results

### 3.1. FE Simulation of Nanoindentation

**Figure 6:**
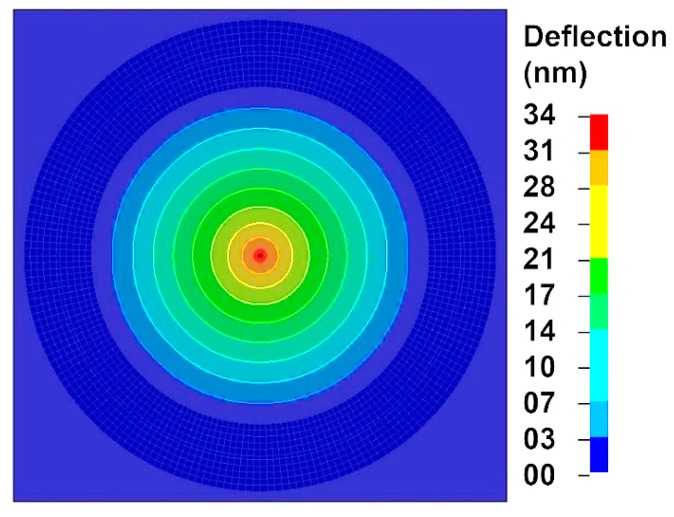
Simulated deflection field of the Ti_3_C_2_T_z_ MXene monolayer.

**Figure 7:**
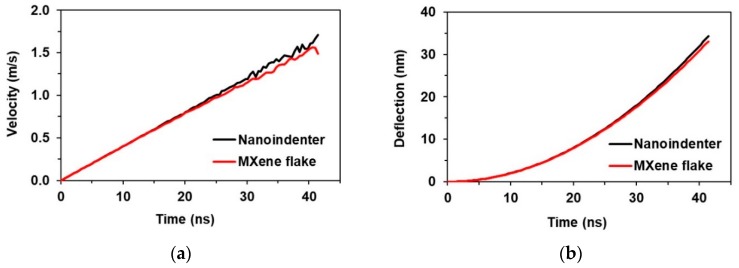
FE results of the nanoindentation simulation: (**a**) velocity of the nanoindenter and the center point of the MXene monolayer flake, (**b**) deflection of the nanoindenter and the center point of the MXene monolayer flake.

The simulated deflection field of the indented MXene monolayer flake is presented in Figure 6. The highest deflection of 34 nm was reached at the center point of the MXene flake. The dependence of velocity of the nanoindenter and the center point of the MXene monolayer flake on time shows a linear behavior (Figure 7a). Analyzing the deflection of the nanoindenter and the center point of the MXene monolayer flake as a function of time, the dependence is observed to be nonlinear (Figure 7b).

**Figure 8:**
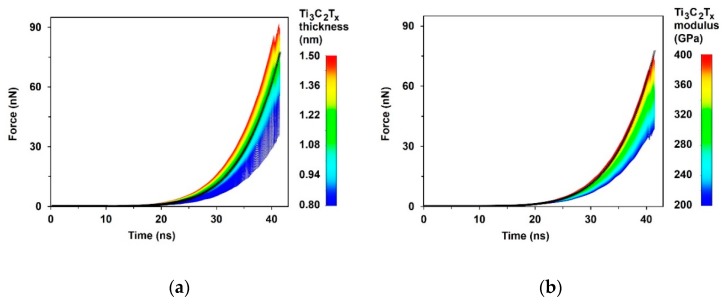
Sensitivity analysis results: (**a**) thickness influence on the force vs. deflection curve when *E* = 333 GPa; (**b**) Young’s modulus influence on the force vs. deflection curve when *h* = 0.98 nm.

The influence of MXene monolayer flake thickness (Figure 8a) and Young’s modulus (Figure 8b) on the force vs. deflection curve was analyzed. The results of thickness influence showed that if the Young’s modulus of the MXene monolayer flake is set as *E* = 333 GPa, the best fit to the experimental result is obtained when the thickness is equal to 1.1 nm, which is higher than results presented in the literature [1,2,11]. On the other hand, if we use a thickness of 0.98 nm, the best fit appears at a Young’s modulus of 380 MPa. To achieve the same results as the experimental ones, for finite element modeling, the thickness of the MXene monolayer flake should be increased from 0.98 to 1.1 nm, or the Young’s modulus should be increased from 333 to 380 GPa.

### 3.2. FE Simulation of Pure MXene Film

**Figure 9:**
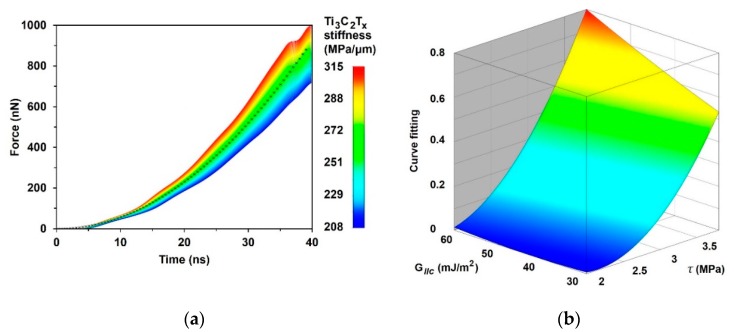
Sensitivity analysis of free-standing three-layer MXene nanosheets: (**a**) force vs. time curve sensitivity upon in-plane stiffness $E_{\mathrm{interl}}$; **×**—experimental results obtained from [4] and curve fitting of MXenes, (**b**) influence of the interlaminar shear strength and shear energy release rate as $E_{\mathrm{interl}}=\;0.26\;\frac{\mathrm{GPa}}{\mu m}$.

To simulate the behavior of pure MXene film under tension conditions, three constants were selected for calibration and sensitivity analysis: interlaminar shear strength, stiffness, and the energy release rate used for damage calculation. The results obtained by the curve fitting approach are shown in Figure 9. The best fitted FE curve was obtained using an interlaminar shear strength $\tau_{\mathrm{interl}}=2.2\;\mathrm{MPa}$, in-plane stiffness $E_{\mathrm{interl}}=0.26\;\frac{\mathrm{GPa}}{\mu m}$, and a shear energy release rate $G_{IIc}\;=\;3.8\times 10^{-2}\;\frac{J}{m^{2}}$. The results show that the behavior of tensioned film of free-standing MXene nanosheets is most sensitive to their interlaminar shear strength and in-plane stiffness, while the shear energy release rate mainly influences the interlaminar damage properties of nanosheets.

**Figure 10:**
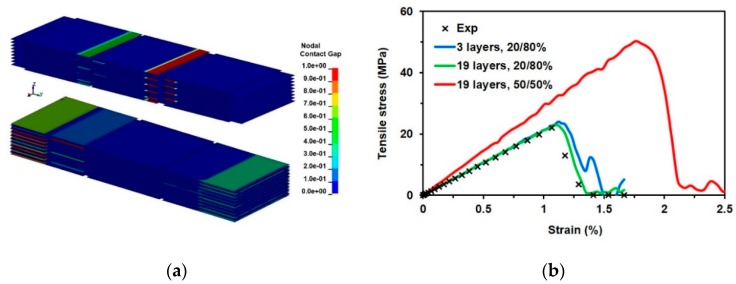
FE simulation results: (**a**) Tiebreak contact gap development episode in cases of 20%/80% and 50%/50% overlap; (**b**) tensile stress–strain curves of different thicknesses for free-standing MXene films; the experimental results are recalculated from the literature [4].

The material parameters identified by FE modeling were based on 20% of the random average overlapping of nanosheets. In the validated FE model, we changed the overlapping of nanosheets by up to 50% and evaluated the behavior of the ideal overlapping case (Figure 10a). By the FE simulations obtained, the force versus displacement relations were transformed into stress–strain curves and compared with the experimental tensile curve [4] (Figure 10b). The simulation results, based on previously identified material parameters and maximum overlapping (50%) compared with the experimental tensile curve, show the increase of the free-standing Ti_3_C_2_T*_z_* film strength, stiffness, and failure strain when the overlapping is increased.

It is clearly seen (Figure 10b, red curve) that the random overlapping of nanosheets decreases the strength and stiffness of films assembled from free-standing MXene nanosheets. The FE models with curve fitting approaches can be used for material constants and adhesion energy identification, and only a proper statistical interpretation of the geometrical parameters of the nanosheets is needed.

## 4. Conclusions

The explicit FE model was developed in LS-Dyna to simulate the nanoindentation process of the 2D Ti_3_C_2_T*_z_* MXene monolayer flake and validate the material model. Sensitivity analysis of Young’s modulus and the flake thickness was performed. The obtained force versus deflection curves showed lower results than the experimental ones. To achieve similar results to the experimental ones, the thickness of the MXene flake should be increased from 0.98 up to 1.1 nm, or the Young’s modulus should be increased from 333 up to 380 GPa.

The reverse engineering method with the curve fitting approach was applied to evaluate the adhesive strength of the assembled free-standing Ti_3_C_2_T*_z_*-based films. The interlaminar shear strength, stiffness, and energy release rate were selected as the main variables; these variables affect the tensile strength of the assembled free-standing Ti_3_C_2_T*_z_*-based film. The experimental tensile test data has been used for the curve fitting approach. A simulation was performed using three and nineteen layers of single-layered nanosheets with 20% overlap. The best fitted FE curve was obtained using an interlaminar shear strength $\tau_{\mathrm{interl}}=\;2.2\;\mathrm{MPa}$, in-plane stiffness $E_{\mathrm{interl}}=0.26\;\frac{\mathrm{GPa}}{\mu m}$, and shear energy release rate $G_{IIc}=\;3.8\times 10^{-2}\;\frac{J}{m^{2}}$. The results of the sensitivity analysis showed that the largest influences on the behavior of tensioned film of free-standing MXene nanosheets are interlaminar shear strength and in-plane stiffness, while the shear energy release rate mainly affects the interlaminar damage properties of nanosheets. The simulation results based on identified material parameters showed an increase in the free-standing Ti_3_C_2_T_z_ film strength, stiffness, and failure strain when the overlapping was increased by 50%.

The developed FE models with curve fitting approaches are of general purpose and can be used to determine material strength and stiffness properties and adhesion energy identification in different cases; however, a proper statistical interpretation of the single-layered nanosheet geometrical parameters is needed.

The obtained values of interlaminar shear strength, stiffness, and energy release rate in MXenes are very important parameters for further investigation of MXene-polymer composites by finite element modeling. Areas for further research include analysis of the influence of multilayered or agglomerated nanosheets on the composite properties as well as development of MXene–polymer coatings with high-density MXene–MXene interactions.

## Figures and Tables

**Table 1:** Mechanical properties of materials used in the simulation of nanoindentation.

Material	Density, ρ, g/cm^3^	Elastic Modulus, *E*, GPa	Poisson’s Ratio, ν	Tensile Strength, σ_u_, GPa
Ti_3_C_2_T_z_	3.19 [8]	333 [8]	0.227 [15]	17.3 [8]
SiO_2_ [16]	2.65	70.0	0.17	-
Diamond nanoindenter	3.50	1000	0.20	-

**Table 2:** The parameters used for the simulation of shear strength between nanosheet interfaces.

Normal Failure Stress, nfls (T), MPa	Shear Failure Stress, sfls (S), Mpa	Normal Energy Release Rates, eraten (G_IC_) mJ/m^2^	Shear Energy Release Rates, erates (G_IIC_) mJ/m^2^	Ratio of Tangential Stiffness to Normal Stiffness, ct2cn	Normal Stiffness, Cn €, MPa/μm
2 ÷ 4	2 ÷ 4	30 ÷ 60	30 ÷ 60	1	200–350
